# Parental self-efficacy, health locus of control, and preventive behaviors against respiratory infections in young children: a cross-sectional survey study

**DOI:** 10.3389/fpubh.2026.1910140

**Published:** 2026-08-18

**Authors:** Anna Bednarek, Marzena Laskowska, Anna Lewandowska, Anna Umińska, Iwona Bodys-Cupak

**Affiliations:** 1 Department of Health Promotion Chair of Nursing Development, Faculty of Health Sciences, Medical University of Lublin, Lublin, Poland; 2 Department of Obstetrics and Perinatology, Faculty of Medicine, Medical University of Lublin, Lublin, Poland; 3 Faculty of Healthcare, State Academy of Applied Sciences in Jaroslaw, Jaroslaw, Poland; 4 University Children’s Hospital in Lublin, Lublin, Poland; 5 Department of Nursing Fundamentals, Faculty of Health Sciences, Institute of Nursing and Midwifery, Jagiellonian University Medical College, Krakow, Poland

**Keywords:** child, GSES scale, infections, MHLC scale - version A, parents, prevention, respiratory system

## Abstract

Respiratory infections in young children are a common health problem influenced by several factors. The aim of this study was to recognition the principles of respiratory tract infection prevention in young children in the context of parents’ self-efficacy and the level of health locus of control. A cross-sectional study was conducted among 150 parents of young children. A proprietary questionnaire and standardized scales were used: the General Self-Efficacy Questionnaire (GSES) and the Multidimensional Health Locus of Control Scale (MHLC – version A). Research material was collected online using Google Forms software. Data from 134 respondents were included in the statistical analysis. A significant correlation was found between the frequency of respiratory infections in children and their age, attendance at nursery or kindergarten, physical activity and suffering from asthma. Based on the GSES sten scores, the majority of parents (66.42%; *n* = 89) had high self-efficacy (sten scores of 7–10). According to the MHLC scale - version A scale, health control was highest on the internal dimension (Me = 26), and chance had the least influence on health control (Me = 20). Parents engaged in health behaviors to prevent respiratory infections in their children. Most parents scored high on the GSES and MHLC scale - version A, which may have translated into better health management skills and the implementation of appropriate health-promoting practices in their children.

## Background

Respiratory infections are a common health problem in young children. They are usually mild upper respiratory tract infections (1). However, lower respiratory tract infections can sometimes occur, which can cause complications and prolong the recovery period. Recurrent infections are particularly unfavorable, negatively affecting the child’s general health and quality of life (2, 3). Additionally, they increase the number of outpatient consultations and can even result in hospitalization and secondary infections in siblings and family (4). Respiratory infections are mainly of viral origin. Depending on the location, the causes may also be bacterial. The most common viral pathogens include rhinoviruses, coronaviruses, adenoviruses, enteroviruses, and respiratory syncytial virus (RSV), as well as parainfluenza and influenza viruses. In the moderate, transitional climate of Europe, viral infections are observed especially in spring and autumn, with the peak incidence in winter. Infections are spread by droplets and by contact with the upper respiratory tract secretions of an infected person. The incubation period is 1–6 days (for most viral and bacterial pathogens), while the period of contagion in the case of infection caused by rhinoviruses can last up to 3 weeks (5, 6). In the case of bacterial infections, the etiological factors are: *Streptococcus pneumoniae*, *Haemophilus influenzae*, *Moraxella catarrhalis*, *Streptococcus pyogenes*, less frequently *Klebsiella pneumoniae*, *Pseudomonas aeruginosa*, *Staphylococcus aureus* and anaerobic bacteria. Among the atypical bacteria responsible for respiratory tract infections, the following are of significant importance: *Mycoplasma pneumoniae*, *Chlamydophila pneumoniae,* and *Legionella pneumophila* (7–9). Respiratory tract infections in children (nose, sinuses, throat, and ears) most often cause nasal discharge and congestion, fever, sore throat or ear. They may be accompanied by a persistent cough (10). Laryngeal and tracheal infections are characterized by a characteristic, persistent “barking” cough, hoarseness, and shortness of breath of varying intensity. In older children and adults, laryngitis may cause hoarseness (11). Bronchitis and pneumonia in children mainly cause fever and cough (12). Recurrent respiratory infections are a common problem in preschool and school-age children. The number of infections may reach up to 10 per year. Recurrent, chronic upper respiratory tract infections in children are considered to be at least three episodes of acute infections in a period of 6 months or from 6 to 8 per year. It is considered that the criteria for recurrent infections are met by an average of 15% of preschool children (8, 13, 14). In children under 5 years of age, the frequency of hospitalization due to acute respiratory infections is 20, and 90% of these cases are pneumonia (6, 12). The most common cause of this disease is RSV infection. Worldwide, every year, children under 5 years of age develop almost 34 million acute lower respiratory tract infections, due to RSV infection alone, with 1/10 children requiring hospitalization (14). The annual number of deaths in early childhood due to acute respiratory infections is estimated at 1.9–2.2 million, with 70% of deaths occurring in developing countries (15). In Poland, infant death rates due to respiratory infections have decreased from 46/100,000 to 1/100,000 in the last 40 years. In 2020, the hospitalization rate due to pneumonia in children aged 1–4 years in Poland was 90.7/10,000 (16). Epidemiological data also indicate that exposure to infection in early life is a significant factor determining the occurrence of immunological diseases in adults (17). Respiratory infections in young children are caused by a number of factors, including genetic, anatomical, and physiological respiratory system, environmental, and family lifestyle factors (1, 7, 18). Individual and environmental factors include: lack of breastfeeding and vitamin supplementation, poor housing conditions (damp, overcrowding), exposure to tobacco smoke (passive smoking impairs natural defense mechanisms and promotes respiratory infections), male gender, prematurity, immunodeficiencies (inborn errors of immunity), chronic lung diseases, and heart defects (19–23).

In preventing respiratory infections, prophylaxis plays a key role, the aim of which is to maintain the child’s good general condition, eliminate risk factors for respiratory infections and secondary infections and complications. First of all, attention should be paid to non- pharmacological methods of treatment that are elements of a healthy lifestyle, whose positive effect on the proper functioning of the immune system has been proven. These include: an adequate amount of sleep, regular and moderate physical exercise, breastfeeding for over 6 months, no exposure to tobacco smoke and air pollution, a rational diet and the associated correct body weight, appropriate housing conditions, complete vaccinations according to the national vaccination program. An important element of prevention is also ensuring that the child stays in an air free from pollution every day (24–26). Immune errors that can promote recurrent upper respiratory tract infections include not only congenital disorders related to cellular and humoral mechanisms, but also, in some cases, acquired disorders, for example, those related to human immunodeficiency virus (HIV) infection in children of seropositive mothers (6, 21). Sometimes, morphological abnormalities are found in children with recurrent and chronic respiratory tract infections. Prevention in these cases involves surgery performed outside the period of infection (2, 19).

Parents should make every effort to take care of their child’s health and safety by eliminating risk factors for respiratory infections. The attitude of parents towards their own health and its promotion is also important (26–28).

Parents play a key role in shaping health-related behaviors in early childhood. Parental self-efficacy, which refers to beliefs and confidence in their ability to promote healthy functioning and support children’s health needs through preventative care, providing healthy foods, and setting limits, is also an important factor in this process. Self-efficacy promotes rational actions of parents regarding their children’s health (29, 30). Moreover, self-confidence correlates with skills and personal success in what one does. The belief that “I can handle the challenge” naturally fosters optimism, curiosity and the will to act (31). Self-efficacy should be treated as a factor influencing the adoption of health-promoting behaviors. The stronger the sense of self-efficacy and the expectations associated with it, the greater the belief in the possibility of achieving good results, lasting effects, and consistent pursuit of a set goal despite encountered obstacles (32).

There are numerous studies that discuss the various determinants of respiratory infections in children and the possibilities of preventing these infections. These infections have a significant impact on the child and family life, and place an economic burden on the family and the healthcare system. Possible causes of respiratory infections in young children include primarily factors related to physiological and immunological differences in children, followed by environmental factors, including parental lifestyle, and sociodemographic factors. These factors likely contribute to the wide variation in infection rates and symptoms observed in early childhood (33). However, to our knowledge, there are no reports that discuss the relationship between parental self-efficacy and health control and the prevention of respiratory infections in young children. This relationship seems to us to be important in supporting the principles of respiratory infection prevention used by parents in their children.

A key mechanism explaining this relationship is self-efficacy, a concept developed by psychologist Albert Bandura (34). It refers to an individual’s belief in their own abilities to organize and effectively carry out activities, in this case, preventive measures against respiratory diseases in their child. This relationship is based on several psychosocial factors, such as initiating and maintaining activities, coping with stress, and modeling behavior. Parents with a high sense of self-efficacy are not only more likely to implement daily hygiene habits but also demonstrate greater perseverance in the face of difficulties in undertaking various preventive activities. Self-confidence protects parents from a sense of helplessness in the case of infectious disease season, allowing them to rationally manage their child’s health and utilize proven and more demanding preventive methods. Parents who are confident in their actions become role models for their children in terms of preventive care (34).

The aim of this study was to recognition the principles of respiratory tract infection prevention in young children in the context of parents’ self-efficacy and the level of health locus of control. The results of our study will fill a gap in the literature in terms of better understanding the role of psychosocial factors in determining parents’ health behaviors toward their children. Preventive actions related to children’s health should pay particular attention to parents’ psychological resources that motivate them to engage in health-promoting activities, including generalized self-efficacy and locus of control over one’s health (35).

## Materials and methods

The study involved 150 parents of small children, aged from birth to 6 years. The inclusion criterion for the study was adult parents (women or men) with a small child (up to 6 years of age). The exclusion criterion for the study concerned minors and people who were not parents of small children.

The sample size was estimated using a sample selection calculator (based on the size of the child population in Poland) and Statistica software (assumptions: maximum error 5%, statistical power M = 0.80).

A self-designed questionnaire and two standardized scales were used as the research tool. The questionnaire of the own survey consisted of 31 questions, including eight questions about the sociodemographic data of the respondents, such as gender, age, place of residence, education, professional activity, marital status, financial status and housing conditions. The remaining 23 questions of the survey concerned obtaining information about the factors determining respiratory infections and their frequency, infection prevention, and family lifestyle. Information about the type of respiratory infection was obtained exclusively from the parents. Participants had this information from the primary care physician who examined the child.

The following scales were also used in the study:

- Generalized Self-Efficacy Scale (GSES) adapted by Schwarzer et al. (36). It presents 10 statements referring to various personal characteristics. After reading each statement, one should decide whether they are true or false concerning oneself. The Cronbach’s alpha coefficient for the Polish version of the GSES is 0.85, which indicates its good internal consistency. This value was obtained based on a study conducted on 174 individuals aged 20–55 years (36).
- Multidimensional Health Locus of Control Scale (MHLC – version A) adapted by Juczyński et al. (37). It presents 18 views of different people on certain important health-related issues. Each statement expresses a view that one can agree with or not. It is important to answer by one’s own beliefs. The author, who adapted the standardized research tool, consented to the use of these scales. In the Polish version of the MHLC scale, Cronbach’s alpha for individual dimensions in version A was 0.74 (internal control), 0.69 (chance), and 0.54 (other). These values indicate acceptable or low scale reliability, especially for the “other” dimension (33, 35).

The participants were asked to respond to the statements on a four-point scale (ranging from “no” – scored as 1 point to “yes” – scored as 4 points). The sum of the points obtained (10–40) determines the overall self-efficacy index, which is then converted to standardized units – sten scores. Interpreting the GSES test results, the participants can be divided into three groups – individuals with high, average, and low self-efficacy. High self-efficacy is experienced by individuals scoring 30 points or higher, corresponding to the 7–10 sten scale; average self-efficacy is experienced by individuals scoring 25–29 points, corresponding to the 5–6 sten scale; and low self-efficacy is experienced by individuals scoring 24 points or lower, corresponding to the 1–4 sten scale (36).

The study material was collected online, using Google Forms software. The Authors assumed a survey period of approximately 4 months. The research tool was posted on the website at the link https://forms.gle/gn4nEUgi3SvaGVeD8, which was created and distributed from February 3 to May 31, 2025, on the Facebook platform, in groups for parents, such as: *Healthy children - Healthy Family*, *Mom for Mom*, *How to help a child in illness*, *Health and illnesses of children*.

Before conducting the main study, a pilot study was conducted on a sample of 12 respondents, using a contact approach, at the University Children’s Hospital in Lublin, in the following clinical departments: the Neonatal Pathology Department, the Infant Pathology Department, and the Department of Pediatrics, Lung Diseases, and Rheumatology. One parent participated in the study. Respondents were provided with a space where they could anonymously complete a survey questionnaire, which included a proprietary tool and two standardized questionnaires, in compliance with GDPR (General Data Protection Regulation) regulations. After collecting the surveys, a preliminary verification of the research tools was conducted. It was found that the instructions were clear to parents, the questions were understandable, and there were no problems with their interpretation. The surveys from the pilot study were included in the analysis of the main study.

Parents participating in the study expressed their conscious, voluntary, written consent to complete an anonymous survey after thoroughly familiarizing themselves with the purpose and scope of the study. The time to complete the questionnaire was approximately 45 min. Respondents could withdraw their consent and withdraw from the study at any stage of completing the online survey without giving a reason.

The study was conducted in accordance with the principles of the Declaration of Helsinki and reported to the Bioethics Committee of the Medical University of Lublin. After reviewing the documentation, the Committee issued an opinion that, in accordance with Polish research law, consent from the Bioethics Committee for the survey study was not required. At the same time, it found no contraindications to continuing the study (KE/108/02/2024).

The database and statistical tests were conducted using STATISTICA 13.0 computer software (StatSoft, Poland). One hundred thirty-four fully completed questionnaires were qualified for the study and statistically analyzed. The reasons for not including 16 questionnaires in further analyses were: withdrawal of consent to the study (5) and incomplete data in the survey (11). The values of the analyzed measurable parameters were presented using the value, mean, and standard deviation, and for the non-measurable ones using the frequency and percentage. For measurable features, the normality of the distribution of the analyzed parameters was assessed using the Shapiro–Wilk W test. For unrelated qualitative features, the Chi^2^ homogeneity test was used to detect the existence of differences between the compared groups. The Chi^2^ independence test was used to examine the existence of relationships between the studied features, and the Mann–Whitney U test was used to compare two independent groups. The Kruskal-Wallis test was used to compare the age of the groups. The relationship between variables was assessed using the Spearman R correlation. The significance level of *p* < 0.05 was adopted, indicating the presence of statistically significant differences or relationships.

## Results

Data from 134 respondents were included in the research analysis. Table 1 characterizes the group of parents in terms of sociodemographic data.

**Table 1:** Characteristics of respondents (*n* = 134).

Category	*N*	%
Gender		
Women	115	85.82
Men	19	14.18
Age range in years		
18–25	30	22.39
26–35	57	42.54
36–45	47	35.07
Place of residence		
City	89	66.42
Rural area	45	33.58
Education		
Higher	82	61.19
Secondary	35	26.12
Vocational	15	11.19
Primary	2	1.49
Professional activity		
Both parents work	95	70.89
One parent works	39	29.10
Status of respondents		
Married	102	76.12
In a civil partnership	23	17.16
Single	8	5.97
Divorced	1	0.75
Family financial situation		
Very good	47	35.07
Good	70	52.24
Average	17	12.69
Family housing conditions		
Very good	78	58.21
Good	56	41.79

The study covered 134 parents, the vast majority of whom were women (85.82%; *n* = 115). The largest group of respondents was people aged 26–35 (42.54%; *n* = 57). The place of residence for most respondents was a city (66.42%; *n* = 89). The respondents most often had higher education (61.19%; *n* = 82) and were professionally active (70.89%; *n* = 95). The majority of respondents were married (76.12%; *n* = 102). The majority of respondents had a good financial status (52.24%; *n* = 70), and (58.21%; *n* = 78) assessed their housing conditions as very good (see Table 2).

**Table 2:** Frequency of respiratory infections in children according to parental age.

Parents’ age	Infection rate		
	Rarely	On average	Together
	*n* %	*n* %	*n* %
18–25 years old	25	5	30
	83.33%	16.67%	100.00%
26–35 years old	34	23	57
	59.65%	40.35%	100.00%
36–45 years old	35	12	47
	74.47%	25.53%	100.00%
Together	94	40	134
	70.15%	29.85%	100.00%

Statistical analysis: Chi^2^ = 5.91; *p* = 0.05.

Children of parents aged 26–35 years suffered from respiratory infections more frequently (Chi^2^ = 5.91; *p* = 0.05) than children of parents aged 18–25 and 36–45 years. The demonstrated correlation is not statistically significant (the differences shown are borderline statistically significant), but it shows a trend in this direction.

### Analysis of own research based on the author’s survey questionnaire

Table 3 summarizes the respondents’ answers regarding the determinants and prevention of respiratory infections in young children.

**Table 3:** Respondents’ answers to the questions in the author’s survey questionnaire (*n* = 134).

Analysis category	*N*	%
Age of children		
3–4 weeks	19	14.18
5 weeks–12 months	20	14.92
13 months–24 months	27	20.15
3–4 years	22	16.42
5–6 years	46	34.33
Biological gender of children		
Boys	74	55.22
Girls	60	44.78
Gestational age at birth		
22–36 weeks of pregnancy	12	8.96
37–41 weeks of pregnancy	98	73.13
42 weeks pregnant	24	17.91
Breastfeeding		
Yes	107	79.85
No	27	20.15
The incidence of chronic diseases in children		
Asthma	15	11.19
Heart defect	4	2.98
Type 1 diabetes	1	0.75
Kidney defects	2	1.49
Child status in the family		
Has no siblings	48	35.83
Has siblings	86	64.18
Child care during the day		
Attends organized group activities/nursery/kindergarten	87	64.92
Remains under the care of parents/family	47	35.07
Incidence of respiratory infections		
Rarely (1–4 times a year)	94	70.15
On average (5–6 times a year)	32	23.88
Often (7–10) times a year	5	3.73
Very often (more than 10 times a year)	3	2.32
Type of respiratory infection*		
Bronchitis	60	40.78
Cold (runny nose, cough, sore throat)	45	33.58
Pneumonia	40	29.85
Otitis media	25	18.66
Pharyngitis	23	17.16
Tonsillitis	18	13.43
Sinusitis	10	7.46
Flu	5	3.73
Whooping cough	3	2.24
Child exposure to second-hand smoke		
Yes	9	6.72
No	125	93.28
Carrying out compulsory vaccinations of children in accordance with the vaccination schedule		
Yes	116	86.57
No	18	13.43
Implementation of recommended vaccinations for children		
Yes	58	43.28
No	76	56.72
Giving your child vitamin D_3_		
Yes	110	82.09
No	24	17.91
Taking care of your child’s hygiene, spending time outdoors and dressing appropriately for the ambient temperature		
Yes	103	76.86
No	31	23.13
Experiencing stressful situations by a child		
Yes	22	16.41
No	112	83.58

*Values do not add up to 100% due to the possibility of selecting multiple answers.

Most children were born full-term (73.13%; *n* = 98), and were breastfeed (79.85%; *n* = 107), mainly up to 6 months of age (32.84%; *n* = 44). Less than 1/3 of children (16.41%; *n* = 22) had a diagnosed chronic disease, most frequently asthma (11.19%; *n* = 15).

The majority of children had siblings (64.18%; *n* = 86) and were under institutional care for various periods of time during the day.

The frequency of respiratory infections was assessed according to the point criterion depending on the number of infections per year (1–4 times per year, 1 point; 5–6 times per year −2 points; 7–10 times points; >10 times points). The mean score for the occurrence of infections was 1.35 ± 0.49 (Me = 1.00; range from 1 to 4). It was shown that respiratory infections occurred in children 1–4 times per year (70.15%; *n* = 94).

The most common infections were bronchitis, colds, and pneumonia. Most infections in the child lasted from 6 to 10 days (44.03%; *n* = 59). The respondents were most likely not smokers (73.88%; *n* = 99) and did not expose their child to passive smoking (93.28%; *n* = 125).

Most of the children were breastfeed (*n* = 107; 79.85%). Non-breastfeed children were more likely to have respiratory infections (40.74%) compared to breastfeed children (27.10%).

Most frequently, surveyed parents (86.57%; *n* = 116) carried out mandatory vaccinations for children according to the national vaccination program, i.e., against hepatitis B, tuberculosis, pneumococci, tetanus, diphtheria, pertussis, poliomyelitis, HIB, mumps, measles, rubella, rotaviruses, and HPV. On the other hand, more than half of parents (56.72%; *n* = 76) did not use recommended vaccinations, i.e., against meningococci, chickenpox, influenza, and COVID-19.

The majority of the respondents gave vitamin D_3_ to their children (82.09%; *n* = 110), usually in a dose of 400 to 600 IU/day, and 17.91% did not supplement vitamin D_3_.

The respondents most often assessed the level of personal hygiene of children as very good (76.86%; *n* = 103) and claimed that children regularly spend time outdoors in air free from pollution and are dressed appropriately for the conditions outside (73.88%; *n* = 99). In addition, they claimed that children are not exposed to stressful situations, regularly sleep through the night, and do not show signs of fatigue (83.58%; *n* = 112).

Children suffering from asthma had significantly more respiratory infections (41.46%) than children who did not have asthma (24.73%); Chi^2^ = 3.801; *p* = 0.05.

Children attending nursery or kindergarten had significantly more respiratory infections than children who did not attend these facilities (Chi^2^ = 14.670; *p* = 0.0001).

Children aged 3–4 years had respiratory tract infections significantly more often (Chi^2^ = 26.670; *p* = 0.00006) than children in other age groups (neonates, infants, children aged 1–2 years and 5–6 years).

Although breastfeed children had fewer respiratory infections than non-breastfeed children, these differences were not statistically significant, Chi^2^ = 1.910; *p* = 0.17.

The study showed that children whose parents maintained average personal hygiene, including dressing appropriately for the ambient temperature and frequenting outdoor activities, had a higher incidence of respiratory infections (40.00%) than children who had excellent personal hygiene (26.26%), dressed appropriately for the ambient temperature, and frequently spent time outdoors. These differences were not statistically significant (Chi^2 =^ 2.33; *p* = 0.13).

Statistical analysis showed that children who led an active lifestyle had significantly more respiratory infections (36.84%) than children who were inactive (20.69%); Chi^2 =^ 4.10; *p* = 0.04.

### Analysis of own research based on the generalized self-efficacy scale (GSES)

The GSES tool, adapted *by* Schwarzer et al. (36), was used to assess self-efficacy. The scale score ranges from 10 to 40 points. The higher the score, the greater the sense of self-efficacy. The average self-efficacy score was 31.43 ± 4.73 (Me = 30.50; range from 21 to 40). The ranked results were converted to sten scores. Table 4 presents the obtained frequencies of occurrence for individual sten scores. The most common scores were within the range of 7 sten scores (30.60%).

**Table 4:** Sten scores for the GSES scale.

Sten	*n*	%	Level of self-efficacy parents	*n*	%
1	0	0.00	Low	6	4.48
2	0	0.00			
3	4	2.99			
4	2	1.49			
5	20	14.93	Average	39	29.10
6	19	14.18			
7	41	30.60	High	89	66.42
8	22	16.42			
9	7	5.22			
10	19	14.17			
Total	134	100.00	Total	134	100.00

The obtained sten scores showed that the majority of parents (66.42%; *n* = 89) had a high level of self-efficacy (7–10 sten), 29.10% (*n* = 39) had an average level (5–6 sten), and 4.48% (*n* = 6) had a low level (1–4 sten).

In the group of parents who had a high level of self-efficacy, respiratory infections in children occurred with a slightly lower frequency (74.16%) than in the group with an average level of self-efficacy (61.54%) and a low level (66.67%). However, the differences shown were not statistically significant (*p* = 0.350). At the same time, respondents with a low sense of self-efficacy more often did not vaccinate their children with mandatory vaccines according to the vaccination schedule (33.33%) compared to respondents with an average level of self-efficacy (12.82%) or a high level (12.36%). The differences shown were not statistically significant (*p* = 0.34). On the other hand, respondents with a high sense of self-efficacy more often assessed that they took very good care of their children’s hygiene (77.53%) than those with an average (69.23%) and low (50%) sense of self-efficacy. The differences shown were not statistically significant (*p* = 0.240).

### Analysis of own research based on the multidimensional health locus of control scale (MHLC - version a)

MHLC – version A scale adapted by Juczyński (37) was used to assess the health locus of control. The scale consists of 18 statements, which are rated from 1 to 6 points. The scale includes three areas of the health locus of control: Internal (In), Influence of others (I) and Case (C). In the study group (Table 5), health control was the highest in the Internal dimension (Me = 26), lower in the dimension – Influence of others (Me = 21), and Case had the least impact on health control (Me = 20).

**Table 5:** Statistical relationships regarding selected variables in the group of parents.

	Infection rate		
	Rarely	Often	Together
	*n* %	*n* %	*n* %
Asthma in a child			
No	70	23	93
	75.27%	24.73%	100.00%
Yes	24	17	41
	58.54%	41.46%	100.00%
Together	94	40	134
	70.15%	29.85%	100.00%
Statistical analysis: Chi^2^ = 3.801; *p* = 0.05			
Kindergarten or nursery			
Yes	32	28	60
	53.33%	46.67%	100.00%
No	62	12	74
	83.78%	16.22%	100.00%
Together	94	40	134
	70.15%	29.85%	100.00%
Statistical analysis: Chi^2^ = 14.67; *p* = 0.0001*			
Child’s age			
Neonates	18	1	19
	94.74%	5.26%	100.00%
Infants	20	0	20
	100.00%	0.00%	100.00%
1–2 years	16	11	27
	59.26%	40.74%	100.00%
3–4 years	9	13	22
	40.91%	59.09%	100.00%
5–6 years	31	15	46
	67.39%	32.61%	100.00%
Together	94	40	134
	70.15%	29.85%	100.00%
Statistical analysis: Chi^2^ = 26.67; *p* = 0.00006*			
Breast-feeding			
Yes	78	29	107
	72.90%	27.10%	100.00%
No	16	11	27
	59.26%	40.74%	100.00%
Together	94	40	134
	70.15%	29.85%	100.00%
Statistical analysis: Chi^2^ = 1.91; *p* = 0.17			
Taking care of the child’s personal hygiene (including dressing appropriately for the ambient temperature and being outdoors)			
Very good	73	26	99
	73.74%	26.26%	100.00%
Average	21	14	35
	60.00%	40.00%	100.00%
Together	94	40	134
	70.15%	29.85%	100.00%
Statistical analysis: Chi^2^ = 2.33; *p* = 0.13			
Active lifestyle of a child			
No	46	12	58
	79.31%	20.69%	100.00%
Yes	48	28	76
	63.16%	36.84%	100.00%
Together	94	40	134
	70.15%	29.85%	100.00%
Statistical analysis: Chi^2^ = 4.10; *p* = 0.04*			

*indicates the statistical significance of the research results.

Tables 6, 7 show only those MHLC – version A scale results that were statistically significant with the analyzed factors influencing respiratory infections in children.

**Table 6:** Health locus of control assessment.

Dimension-control	M	Me	Q1	Q3	SD
Internal (In)	25.91	26.00	22.00	30.0	5.01
Influence of others (I)	20.37	21.00	16.00	25.00	6.68
Case (C)	19.30	20.00	14.00	25.00	6.79

**Table 7:** Assessment of health locus of control concerning the scope of personal hygiene care in children.

Dimension- control	Very good			On average			Z	*p*
	M	Me	SD	M	Me	SD		
Internal (In)	26.44	27.00	4.33	24.40	25.00	6.41	−1.83	0.070
Influence of others (I)	21.85	23.00	5.99	16.17	16.00	6.85	−4.35	0.00001*
Case (C)	20.81	22.00	6.48	15.03	15.00	5.84	−4.36	0.00001*

Parents who were very concerned about their children’s personal hygiene had a significantly higher sense of health control in the Influence of others (*p* = 0.00001) and Case (*p* = 0.00001) dimensions than parents who were average in their children’s personal hygiene (Table 7). The internal health control dimension was also slightly higher in the group of respondents who were very concerned about their children’s hygiene than in parents who were average in their children’s hygiene. However, these differences were not statistically significant (*p* = 0.070).

Respondents who did not vaccinate their children (Table 8) had a statistically significantly higher Internal Health Control dimension than parents who vaccinated their children (*p* = 0.010). Respondents who did not vaccinate their children had a slightly higher level of health control in the dimension of Case, and in vaccinated individuals in the Influence of others dimension. However, no significant differences were found in the assessment of the levels of these health dimensions between the groups (*p* > 0.050).

**Table 8:** Assessment of health locus of control concerning the implementation of compulsory vaccinations in children.

Dimension-control	Yes			No			Z	*p*
	M	Me	SD	M	Me	SD		
Internal (In)	24.52	24.00	5.34	26.97	27.00	4.50	−2.55	0.010*
Influence of others (I)	20.90	21.00	6.63	19.96	21.00	6.74	0.67	0.500
Case (C)	18.14	18.00	6.65	20.18	22.00	6.81	−1.71	0.091

*indicates the statistical significance of the research results.

## Discussion

Respiratory infections are common in young children, and their pathogenesis is complex and involves many factors (23). These include the anatomy and physiology of the respiratory system, vitamin or trace element deficiencies, genetic and environmental factors, and impaired immunity (24). Both the upper and lower respiratory tracts can become infected, and recurrent lower respiratory tract infections, such as pneumonia, remain the leading cause of infection in children under 5 years of age (18).

Respiratory infections can lead to serious health complications, especially in children whose immune system is still underdeveloped (31). Identification of risk factors and methods of preventing infections is important in maintaining the health of the child, therefore, this study aimed to recognition the principles of prevention of respiratory infections in young children in the context of the assessment of self-efficacy and the level of health locus of control by parents. The final statistical analysis of the study results included responses obtained from 134 parents, the majority of whom were women (85.82%) and parents aged 26–35 (42.54%), with higher education (61.19%).

Due to the cross-sectional nature of our study, in the detailed analyses and summaries, we focused on the general connections between our results and reports in the medical literature without delving into the underlying causes of our data.

One of the most common pathogens causing acute respiratory infections in young children, which are the cause of hospitalization, is RSV. Worldwide, this virus is responsible for 33 million infections and over 3.5 million hospitalizations (38, 39). Recurrent respiratory infections in developed countries occur in 25% of children under 1 year of age and 18% of children aged 1–4 years (18). In a study conducted by Kurt et al. (31), it was shown that more than half of the parents of children who attended a nursery (61.7%) indicated frequent respiratory infections, also in the last 2 weeks. In addition, the vast majority of children (91.5%) received antibiotics. During the study conducted by the authors, the number of COVID-19 cases was high worldwide, but the fact that parents did not report COVID-19 cases in children could be related to the rarely observed and mild symptoms of this disease in children (39, 40).

Our studies have shown that respiratory tract infections were most common in children aged 1 to 4 (36.57%) and 5–6 years (34.33%), while newborns (14.18%) and infants (14.92%) suffered from them rarely.

Factors such as parental smoking and asthma may contribute to the development of recurrent respiratory infections in children, but no consensus has been reached (41–43). Studies by Vardabas et al. (44) have shown significant associations between parental smoking and respiratory infections. In turn, Zhuge et al. (45) found that parental smoking was not associated with respiratory infections in children, while the smell of indoor smoke was a more direct indicator of exposure to respiratory infections. Observations by Qian et al. (46) showed an association between dry cough and the feeling of stuffy, unpleasant odor, moist or dry air, and mold. Parental smoking is one of the identified risk factors for childhood pneumonia, while passive smoking increases the risk of asthma exacerbation in children (41, 43). Very often, parents do not smoke in the presence of children, but smoking on the balcony or near a window when children are not present may also cause smoke to enter the room, which affects the exposure of children to toxic substances resulting from smoking (43).

In our study, the majority of parents did not smoke cigarettes (73.88%), and 93.28% of respondents stated that they did not expose their children to passive inhalation of tobacco, which we believe is a positive phenomenon and may indicate parents’ health awareness of the harmful effects of smoking.

The influence of asthma on the development of recurrent respiratory infections is a subject of much discussion. Asthma or other chronic diseases may cause recurrent respiratory infections, e.g., sinusitis, rhinitis, otitis media (21, 22, 43). Population studies show that in children with asthma, upper and lower respiratory tract infections occur more often and last longer than in children without allergies and asthma (38, 44). Similarly, in our study, we observed that children with asthma had significantly more respiratory infections (41.46%) than children who did not have asthma (24.73; Chi^2^ = 3.80; *p* = 0.050).

Breast milk contains many protective factors, such as immunoglobulins, lactoferrin and lymphocytes, which may contribute to reducing infections in infants. Breastfeeding also plays a protective role in respiratory infections, and this role becomes more dominant with increasing breastfeeding duration (47). Our study showed that breastfeed children (27.10%) were less likely to develop respiratory infections (40.74%) than non-breastfeed children, which may be due to antibodies contained in breast milk that protect against infections.

In recent years, there has been growing interest in the potential role of vitamin D_3_ in preventing respiratory infections (48). Vitamin D has immunomodulatory and anti- inflammatory effects, hence some reports confirm a significant decrease in the number of respiratory infections in children receiving vitamin D_3_ supplementation. However, the results of some studies in this area are inconclusive. Furthermore, the dosage and duration of vitamin D_3_ supplementation varied across studies (48, 49). In a systematic review, Martineau et al. (50) demonstrated that in observational studies, individuals with severe vitamin D deficiency who received daily or weekly supplementation without additional bolus doses experienced greater health benefits.

Statistical analysis of our study showed that the majority of respondents gave their children vitamin D_3_ (82.09%; *n* = 110), usually at a dose of 400 to 600 IU/day. However, we found that children who did not receive vitamin D_3_ supplementation (17.91%; *n* = 24) had slightly more respiratory tract infections (36%) than children who received vitamin D_3_ supplementation (28.44%).

Preschool facilities that provide care for children, where they have close contact and social interactions, may be the cause of an increased risk of respiratory infections in children. In addition, greater exposure to infectious agents results from the immaturity of the immune system at this stage of life and insufficient hygiene (11–13, 50). Therefore, one of the important preventive measures is to improve the environmental conditions in which children live, ensuring good hygiene, proper nutrition, and physical activity (1, 15, 22). It is also necessary to prevent infections through the use of protective vaccinations, which can reduce the burden of infection with some pathogens, the number of prescribed antibiotics, and hospitalizations due to complications in children and adolescents (5, 6, 8, 9, 51). In our study, we found that children attending preschool or daycare were more likely to have respiratory infections (46.67%) than those who did not attend these facilities (16.22%). At the same time, children whose parents maintained average personal hygiene had slightly more respiratory infections (40%) than those with excellent personal hygiene (26.26%). Furthermore, we observed that children with active lifestyles were more likely to have respiratory infections (36.84%) than those who were inactive (20.69%). This may be explained by the fact that physically active children have greater contact with peers, which increases the risk of infection. In addition, the respiratory system of young children is more susceptible to infections due to its immaturity.

Our research has shown that most parents vaccinated their children according to the vaccination schedule (86.57%), and 43.28% of surveyed parents also used recommended vaccinations. Children who were not vaccinated with mandatory vaccines had respiratory infections slightly more often (44.44%) than children vaccinated according to the vaccination schedule (27.59%). Also, children who were not vaccinated with recommended vaccines had respiratory infections slightly more often (75%) than vaccinated children (63.79%).

According to the assumptions of the social cognitive theory, the sense of self-efficacy is an element of control over personal action. The higher it is, the more strongly an individual is involved in the intended behavior, even in situations when they encounter obstacles and experience failures. The sense of self-efficacy allows for predicting intentions and actions in various areas of human activity, especially in the field of health behaviors (32, 33).

In our studies, it found that the majority of parents had a high level of self-efficacy (66.42%). Moreover, in parents who had a high level of self-efficacy, respiratory infections in their children occurred less frequently (74.16%) than in the group with an average (61.54%) or low (66.67%) level of self-efficacy. It was also shown that parents with a high sense of self-efficacy more often vaccinated their child with recommended vaccines (43.82%), gave vitamin D_3_ (85.39%), took care of the child’s active lifestyle (59.55%), and took very good care of their hygiene (77.53%).

A sense of health locus of control means believing in the impact of health-promoting behaviors on an individual’s health and taking actions to protect or improve it.

There are external and internal loci of control. The first assumes that events are determined by factors beyond our control, such as the actions of other people, fate, or chance, while the second involves perceiving the situation as the result of one’s actions. People with an internal locus of control take responsibility for their actions, seek information, and make autonomous decisions (32, 37). Conscious health control is directly related to the sense of responsibility for one’s health (52).

Our research has shown that in the studied group, health control was the highest in the internal dimension, while lower in the dimension – influence of others, and chance had the smallest influence on health control. It was also found that parents who took very good care of their child’s hygiene had health control located in the external dimension to a higher degree.

### Limitations

Our study has several limitations. Primarily, this study was cross-sectional in nature, meaning its results only allow for inferences about the co-occurrence of specific phenomena, but do not allow for a cause-and-effect analysis of the relationships between them. This also limits the assessment of the dynamics of parents’ health behaviors toward their children, as data were collected at a single, specific point in time. Therefore, we cannot determine whether high self-efficacy and an internal locus of control for parents’ health actually determine the use of respiratory infection prevention principles in children. Furthermore, self-efficacy and the level of control over health are dynamic traits that can change in response to parents’ life experiences, coping with a child’s illness, or stress. A cross-sectional study treats these phenomena as constants, limiting the possibility of drawing conclusions about their impact.

Furthermore, our study included only a small sample of parents of young children from the European population. Furthermore, validation of the research tools for the study group was not conducted, which should be undertaken in the future. Our study sample, although conveniently selected through online parenting groups, provided only self-report data without clinical verification and without ascertaining the family’s financial status and living arrangements. The study relied solely on an online survey. Participants were parents of young children, who typically have limited time to complete surveys due to childcare demands. Another limitation of our survey study may have been “memory bias,” as some of the information obtained from parents required recall of past events related to their children’s health. Furthermore, survey questionnaires are based on parents’ subjective perceptions. Parents may tend to provide responses deemed socially desirable, idealizing their daily preventive and hygiene habits. Therefore, further research with larger samples and in-depth analyses using qualitative data are necessary to better understand the prevention of respiratory infections in young children and identify interventions that support parental education.

Despite these limitations, the value of our study lies in its originality. No reports were found in the literature examining the relationship between parents’ self-efficacy and level of control over their health and the prevention of respiratory infections in their young children. The results of this study also have application value. They can be used in practice, particularly by nurses, to educate parents about prevention and health promotion.

## Conclusion

When presenting the results of our study, it is important to note that the sample of parents studied was small, and the data obtained were based solely on self-reported information and did not include clinical or verified sociodemographic data. Furthermore, most of our results were not statistically significant, although they did indicate trends in this direction. Parents most frequently took actions to prevent respiratory infections in their children, including vaccinations according to the vaccination schedule and recommended vaccinations, vitamin D3 supplementation, maintaining their child’s personal hygiene, spending regular time outdoors, and avoiding exposure to tobacco smoke. Respiratory infections in our study occurred primarily in children aged 1–4 years, and children attending preschool or daycare were at greater risk of developing respiratory infections. It is likely that higher levels of parental self-efficacy and health control may have influenced some of their actions to prevent respiratory infections in young children.

### Research implications for nursing practice

Nurses, in particular, should effectively strengthen the prevention of respiratory infections in children by building parental awareness of home hygiene and promoting a healthy lifestyle. Before beginning parent education, it is important to determine the level of knowledge and educational gaps in family members. Based on this, individualized educational plans can be developed for each family. These should include teaching the entire family proper handwashing and disinfection techniques. Education should also include regular cleaning of touch surfaces and airing rooms to reduce pathogen concentrations and implementing strategies to limit infection transmission. Providing parents with accurate information about the schedule of mandatory and recommended vaccinations is also crucial. Furthermore, counseling on a balanced, vitamin-rich diet, outdoor physical activity, and raising parents’ awareness of the harmful effects of tobacco smoke (including passive smoking), which directly weakens children’s respiratory systems, should be provided.

Our results may have implications for health promotion and health education through the development of counseling networks to more effectively reach parents who struggle to take preventive measures for their children. Parents should be continuously educated on how to prevent respiratory infections in children and how to take care of their own health. A key role in this education should be played by all members of the healthcare team, especially doctors, nurses, and midwives, who interact with parents and their children in various settings. Health education is a powerful tool that equips individuals with the knowledge and skills necessary to make informed health decisions. Well-thought-out educational content on prevention bridges the gap between scientific knowledge and practical application in everyday life. However, further comprehensive research in this area involving other, representative groups is necessary.

## Data Availability

The original contributions presented in the study are included in the article/supplementary material, further inquiries can be directed to the corresponding author.

## References

[1] Zhu G Xu D Zhang Y Wang T Zhang L Gu W . Epidemiological characteristics of four common respiratory viral infections in children. Virol J. (2021) 18:10. doi: 10.1186/s12985-020-01475-y 33407659 PMC7787583

[2] Choi E Ha KS Song DJ Lee JH Lee KC. Clinical and laboratory profiles of hospitalized children with acute respiratory virus infection. Korean J Pediatr. (2018) 61:180–6. doi: 10.3345/kjp.2018.61.6.180, 29963101 PMC6021362

[3] Barros PB Xavier LF Herter EDC Fernandes MFGM Ferreira ICS Pinto LA. Atypical bacterial respiratory infections in children. J Bras Pneumol. (2024) 50:e20240126. doi: 10.36416/1806-3756/e20240126, 38808838 PMC11185144

[4] Correia W Dorta-Guerra R Sanches M Valladares B de Pina-Araújo IIM Carmelo E. Epidemiological and clinical profile of viral respiratory infections in children under 5 years at pre- and post-COVID-19 era in Praia, Cabo Verde. Trop Med Int Health. (2025) 30:694–703. doi: 10.1111/tmi.14125, 40390559 PMC12213327

[5] Furman D Campisi J Verdin E Carrera-Bastos P Targ S Franceschi C . Chronic inflammation in the etiology of disease across the life span. Nat Med. (2019) 25:1822–32. doi: 10.1038/s41591-019-0675-0, 31806905 PMC7147972

[6] Green CA Sande CJ de Lara C Thompson AJ Silva-Reyes L Napolitano F . Humoral and cellular immunity to RSV in infants, children and adults. Vaccine. (2018) 36:6183–90. doi: 10.1016/j.vaccine.2018.08.056, 30177258

[7] Rueda ZV Aguilar Y Maya MA López L Restrepo A Garcés C . Etiology and the challenge of diagnostic testing of community-acquired pneumonia in children and adolescents. BMC Pediatr. (2022) 22:169. doi: 10.1186/s12887-022-03235-z, 35361166 PMC8968093

[8] Thornton HV Blair PS Lovering AM Muir P Hay AD. Clinical presentation and microbiological diagnosis in paediatric respiratory tract infection: a systematic review. Br J Gen Pract. (2015) 65:e69–81. doi: 10.3399/bjgp15X683497, 25624310 PMC4325442

[9] Murgia V Manti S Licari A de Filippo M Ciprandi G Marseglia GL. Upper respiratory tract infection-associated acute cough and the urge to cough: new insights for clinical practice. Pediatric Allergy Immunology and Pulmonology. (2020) 33:3–11. doi: 10.1089/ped.2019.1135, 33406022 PMC7875114

[10] Principi N Autore G Ramundo G Esposito S. Epidemiology of respiratory infections during the COVID-19 pandemic. Viruses. (2023) 15:1–13. doi: 10.3390/v15051160, 37243246 PMC10224029

[11] Henares D Brotons P de Sevilla MF Fernandez-Lopez A Hernandez-Bou S Perez-Argüello A . Differential nasopharyngeal microbiota composition in children according to respiratory health status. Microb Genom. (2021) 7:000661. doi: 10.1099/mgen.0.000661, 34699345 PMC8627214

[12] Wang J Wang R Xie Z. Rethinking paediatric respiratory infections: the role of mixed pathogen infections. Rev Med Virol. (2025) 35:e70021. doi: 10.1002/rmv.70021, 40000823

[13] Asner SA Science ME Tran D Smieja M Merglen A Mertz D. Clinical disease severity of respiratory viral co-infection versus single viral infection: a systematic review and meta-analysis. PLoS One. (2014) 9:e99392. doi: 10.1371/journal.pone.0099392, 24932493 PMC4059637

[14] Priante E Cavicchiolo ME Baraldi E. RSV infection and respiratory sequelae. Minerva Pediatrics. (2018) 70:623–33. doi: 10.23736/S0026-4946.18.05327-6, 30379052

[15] DeJonge PM Monto AS Malosh RE Petrie JG Callear A Segaloff HE . Comparing the etiology of viral acute respiratory illnesses between children who do and do not attend childcare. Pediatr Infect Dis J. (2023) 42:443–8. doi: 10.1097/INF.0000000000003884, 36854108

[16] Góra D Michałek-Piernik B. Elaboration of selected respiratory system diseases in children in the pediatric hospital in Bielsko-Biala in years 2015-2022. Przegląd Epidemiologiczny. (2024) 78:56–68. doi: 10.32394/pe.77.49, 38904312

[17] Schaad UB Esposito S Razi CH. Diagnosis and management of recurrent respiratory tract infections in children: a practical guide. Arch Pediatr Infect Dis. (2016) 4:e31039. doi: 10.5812/pedinfect.31039

[18] Thanyawee P Suvaporn A Watsamon J. Prevention of emerging infections in children. Pediatr Clin N Am. (2022) 69:185–202. doi: 10.1016/j.pcl.2021.08.006, 34794674 PMC8592647

[19] Mahmood L Flores-Barrantes P Moreno LA Manios Y Gonzalez-Gil EM. The influence of parental dietary behaviors and practices on children's eating habits. Nutrients. (2021) 13:1138. doi: 10.3390/nu13041138, 33808337 PMC8067332

[20] Jesenak M Urbancikova I Banovcin P. Respiratory tract infections and the role of biologically active polysaccharides in their management and prevention. Nutrients. (2017) 9:779. doi: 10.3390/nu9070779, 28726737 PMC5537893

[21] Wong VWY Cowling BJ Aiello AE. Hand hygiene and risk of influenza virus infections in the community: a systematic review and meta-analysis. Epidemiol Infect. (2014) 142:922–32. doi: 10.1017/S095026881400003X, 24572643 PMC4891197

[22] Marengo R Martell JAO Esposito S. Paediatric recurrent ear, nose and throat infections and complications: can we do more? Infect Dis Ther. (2020) 9:275–90. doi: 10.1007/s40121-020-00289-3, 32333286 PMC7237599

[23] Zhang J Sun RR Yan ZX Yi WX Yue B. Correlation of serum vitamin a, D, and E with recurrent respiratory infection in children. Eur Rev Med Pharmacol Sci. (2019) 23:8133–8. doi: 10.26355/eurrev_201909_19033, 31599442

[24] Van Arragon M Grant CC Scragg RK Jordan VMB. Vitamin D for preventing acute respiratory infections in children up to five years of age. Cochrane Database Syst Rev. (2026) 2022:CD015111. doi: 10.1002/14651858.cd015111, 42037591 PMC13112518

[25] Norhayati MN Ho JJ Azman MY. Influenza vaccines for preventing acute otitis media in infants and children. Cochrane Database Syst Rev. (2015) 3:CD010089. doi: 10.1002/14651858.cd010089 25803008

[26] Kuchar E Karlikowska-Skwarnik M Szenborn L Jackowska T Doniec Z Mastalerz-Migas A. Recommendations for primary healthcare doctors for the management of acute respiratory infections in children during the SARS-CoV-2 pandemic – COVID COMPASS. Fam Med Prim Care Rev. (2021) 23:116–24. doi: 10.5114/fmpcr.2021.102647

[27] Carroll FE Al-Janabi H Rooshenas L Owen-Smith A Hollinghurst S Hay AD. Parents’ preferences for nursery care when children are unwell: a discrete choice experiment. J Public Health (Oxf). (2020) 42:161–8. doi: 10.1093/pubmed/fdy215 30576558

[28] McEvoy CT Spindel ER. Pulmonary effects of maternal smoking on the fetus and child: effects on lung development, respiratory morbidities, and life long lung health. Paediatr Respir Rev. (2017) 21:27–33. doi: 10.1016/j.prrv.2016.08.005, 27639458 PMC5303131

[29] Derwig M Tiberg I Björk J Kristensson HI. Changes in perceived parental self-efficacy after a child-Centred health dialogue about preventing obesity. Acta Paediatr. (2022) 111:1956–65. doi: 10.1111/apa.16453, 35702925 PMC9543087

[30] Albanese AM Russo GR Geller PA. The role of parental self-efficacy in parent and child well-being: a systematic review of associated outcomes. Child Care Health Dev. (2019) 45:333–63. doi: 10.1111/cch.12661, 30870584

[31] Kurt G Serdaroğlu HU. Prevalence of infectious diseases in children at preschool education institutions and stakeholder opinions. Children (Basel). (2024) 11:447. doi: 10.3390/children11040447, 38671664 PMC11049231

[32] Jeong J Franchett EE Ramos de Oliveira CV Rehmani K Yousafzai AK. Parenting interventions to promote early child development in the first three years of life: a global systematic review and meta-analysis. PLoS Med. (2021) 18:e1003602. doi: 10.1371/journal.pmed.1003602 33970913 PMC8109838

[33] Sebri V Cincidda C Pravettoni G. Perceived self-efficacy in decision-making: the influence of personality and cognitive variables. Psychooncology. (2025) 34:e70322. doi: 10.1002/pon.70322, 41161817 PMC12571587

[34] Bandura A Barbaranelli C Caprara GV Pastorelli C. Self-efficacy beliefs as shapers of children’s aspirations and career trajectories. Child Dev. (2001) 72:187–206. doi: 10.1111/1467-8624.00273, 11280478

[35] Kwok C Waller D Kohn M Doyle FL. The impact of perceived self-efficacy on healthcare transition outcomes: perceptions from parents and young people. Child Care Health Dev. (2025) 51:e70125. doi: 10.1111/cch.70125, 40562061 PMC12196554

[36] Schwarzer R Jerusalem M. The general self-efficacy scale (GSES). Anxiety Stress Coping. (2010) 12:329–45.

[37] Juczyński Z. Self-efficacy - theory and measurement. J Acta Univ Lodz Folia Psychol. (2000) 4:11–24.

[38] Li Y Wang X Blau DM Caballero MT Feikin DR Gill CJ . Global, regional, and national disease burden estimates of acute lower respiratory infections due to respiratory syncytial virus in children younger than 5 years in 2019: a systematic analysis. Lancet. (2022) 399:2047–64. doi: 10.1016/S0140-6736(22)00478-0, 35598608 PMC7613574

[39] Lange J Kozielski J Bartolik K Kabicz P Targowski T. Analysis of the incidence of acute respiratory diseases in the paediatric population in Poland in the light of the “health needs map”. Adv Respir Med. (2020) 88:204–14. doi: 10.5603/ARM.2020.0106, 32706104

[40] World Health Organization. Coronavirus disease (COVID-19). Available online at: https://www.who.int/emergencies/diseases/novel-coronavirus-2019/situation-reports.

[41] Wada T Adachi Y Murakami S Ito Y Itazawa T Tsuchida A . Maternal exposure to smoking and infant's wheeze and asthma: Japan environment and children’s study. Allergol Int. (2021) 70:445–51. doi: 10.1016/j.alit.2021.04.008, 34140239

[42] Miyahara R Takahashi K Thi Hien Anh N Anh NTH Thiem VD Suzuki M . Exposure to paternal tobacco smoking increased child hospitalization for lower respiratory infections but not for other diseases in Vietnam. Sci Rep. (2017) 7:45481. doi: 10.1038/srep45481, 28361961 PMC5374438

[43] Zhou B Niu W Liu F Yuan Y Wang K Zhang J . Risk factors for recurrent respiratory tract infection in preschool-aged children. Pediatr Res. (2021) 90:223–31. doi: 10.1038/s41390-020-01233-4, 33173178

[44] Vardavas CI Hohmann C Patelarou E Martinez D Henderson AJ Granell R . The independent role of prenatal and postnatal exposure to active and passive smoking on the development of early wheeze in children. Eur Respir J. (2016) 48:115–24. doi: 10.1183/13993003.01016-2015, 26965294

[45] Zhuge Y Qian H Zheng X Huang C Zhang Y Zhang M . Residential risk factors for childhood pneumonia: a cross-sectional study in eight cities of China. Environ Int. (2018) 116:83–91. doi: 10.1016/j.envint.2018.03.022, 29654951

[46] Qian H Zheng X Zhang M Weschler L Sundell J. Associations between parents’ perceived air quality in homes and health among children in Nanjing, China. PLoS One. (2016) 11:e0155742. doi: 10.1371/journal.pone.0155742, 27191186 PMC4871534

[47] Lyons KE Ryan CA Dempsey EM Ross RP Stanton C. Breast milk, a source of beneficial microbes and associated benefits for infant health. Nutrients. (2020) 12:1039. doi: 10.3390/nu12041039 32283875 PMC7231147

[48] Jadhav S Khanwelkar C Jadhav A Seshla S. Vitamin D supplementation in the prevention of recurrent acute respiratory tract infections in children aged <5 years. J Med Sci. (2021) 41:129–33. doi: 10.4103/jmedsci.jmedsci_101_20

[49] Xiao J He W. The immunomodulatory effects of vitamin D drops in children with recurrent respiratory tract infections. Am J Transl Res. (2021) 13:1750–6. 33841698 PMC8014391

[50] Martineau AR Jolliffe DA Hooper RL Greenberg L Aloia JF Bergman P . Vitamin D supplementation to prevent acute respiratory tract infections: systematic review and meta-analysis of individual participant data. BMJ. (2017) 356:i6583. doi: 10.1136/bmj.i6583, 28202713 PMC5310969

[51] Søegaard SH Spanggaard M Rostgaard K Kamper-Jørgensen M Stensballe LG Schmiegelow K . Childcare attendance and risk of infections in childhood and adolescence. Int J Epidemiol. (2023) 52:466–75. doi: 10.1093/ije/dyac219, 36413040

[52] Rosińska P. Poczucie własnej skuteczności i lokalizacja kontroli zdrowia jako predyktory troski o zdrowie w grupie matek małych dzieci. Kwart Nauk Fides Et Ratio. (2017) 32:113–42.

